# Intra-Individual Comparison of Sinus and Ectopic Beats Probing the Ventricular Gradient’s Activation Dependence

**DOI:** 10.3390/jcdd10020089

**Published:** 2023-02-20

**Authors:** Resi M. Schoonderwoerd, Mariëlle Dik, Sumche Man, Arie C. Maan, J. Wouter Jukema, Cees A. Swenne

**Affiliations:** 1 Cardiology Department, Leiden University Medical Center, Albinusdreef 2, 2333 ZA Leiden, The Netherlands; 2 Netherlands Heart Institute, Moreelsepark 1, 3511 EP Utrecht, The Netherlands

**Keywords:** action potential duration restitution, extrasystoles, intraventricular conduction, spatial ventricular gradient, vectorcardiogram

## Abstract

Wilson assumed that the ventricular gradient (VG) is independent of the ventricular activation order. This paradigm has often been refuted and was never convincingly corroborated. We sought to validate Wilson’s concept by intra-individual comparison of the VG of sinus beats and ectopic beats, thus assessing the effects of both altered ventricular conduction (caused by the ectopic focus) and restitution (caused by ectopic prematurity). We studied standard diagnostic ECGs of 118 patients with accidental extrasystoles: normally conducted supraventricular ectopic beats (SN, N = 6) and aberrantly conducted supraventricular ectopic beats (SA, N = 20) or ventricular ectopic beats (V, N = 92). In each patient, we computed the VG vectors of the predominant beat, $\vec{VG_{p}}$, of the ectopic beat, $\vec{VG_{e}}$, and of the VG difference vector, $\vec{\Delta VG_{ep}}$, and compared their sizes. $\left|\vec{VG_{e}}\right|$ of the SA and V ectopic beats were significantly larger than $\left|\vec{VG_{p}}\right|$ (53.7 ± 25.0 vs. 47.8 ± 24.6 mV∙ms, respectively; *p* < 0.001). $\left|\vec{\Delta VG_{ep}}\right|$ were three times larger than the difference of $\left|\vec{VG_{e}}\right|$ and $\left|\vec{VG_{p}}\right|$ (19.94 ± 9.76 vs. 5.94 mV∙ms, respectively), demonstrating differences in the $\vec{VG_{p}}$ and $\vec{VG_{e}}$ spatial directions. The amount of ectopic prematurity was not correlated with $\left|\vec{\Delta VG_{ep}}\right|$, although the larger VG difference vectors were observed for the more premature (<80%) extrasystoles. Electrical restitution properties and electrotonic interactions likely explain our findings. We conclude that the concept of a conduction-independent VG should be tested at equal heart rates and without including premature extrasystoles.

## 1. Introduction

The ventricular gradient (VG), defined as the integral of the ECG amplitude over the QT interval, has intrigued many investigators since it was introduced by Wilson et al. in 1931 [1]. Originally defined for separate ECG leads, the VG evolved into a three-dimensional concept—the spatial ventricular gradient [2]—when vectorcardiography emerged (see Man et al. [3] for an introduction to vectorcardiography). From the beginning, the VG was considered to be independent of the intraventricular conduction order, only depending on “local variations in the excitatory process” [1,4], i.e., on action potential heterogeneity. Hence, the VG was considered individual intrinsic property of the ventricular myocardium.

Our here-described work aims to further contribute to verifying the paradigm of an activation-independent VG. The following introduction discusses the concept of the VG and defines the electrophysiological mechanisms that play a key role in experiment design and in interpreting the observations made by both others and ourselves in order to test the concept of an activation-independent VG.

### 1.1. Vectorcardiographic Concept of the Ventricular Gradient

In 1957, Burger mathematically derived a formula for the VG in 3D vectorcardiographic space. He showed that VG, computed as the 3D integral of the heart vector over the QT interval, is proportional to the volume integral of the 3D action potential duration gradients over the heart [5,6]. This can be summarized in the following two equations (in the original publication numbered 1 and 18, respectively):

(1) $$\vec{G}=\int_{QT}\vec{H}\cdot dt=-\int_{vol}\vec{\nabla }\left(h\cdot \tau \right)\cdot dv$$

where:

$\vec{G}$ = the spatial ventricular gradient vector (VG);

$\vec{H}$ = the heart vector;

h = the potential jump at the boundary of excited and non-excited tissue (i.e., action potential amplitude);

τ = the time lag of repolarization and depolarization (i.e., action potential duration);

$\vec{\nabla }$ = spatial gradient operator (three-dimensional differential, first derivative in the vectorcardiographic X, Y, and Z directions).

### 1.2. QRS Integral, T Integral, QRS Axis, T Axis, Spatial QRS-S Angle

In the same publication, Burger pointed out that the ventricular gradient (according to the formula, the spatial area covered by the course of the heart vector during the QT interval, briefly called the QT integral) equals the sum of the areas covered by the course of the heart vector during the QRS complex and during the T wave, briefly called the QRS integral and the T integral, respectively.

Like the QT integral, the QRS and T integrals are vectors, of which the spatial directions identify the QRS and T axes, respectively. The angle between the QRS and T integrals is the QRS-T angle. The relations between the QRS and T integrals, the QRS-T angle, and the ventricular gradient can be associated with specific ECG phenomena like concordance/discordance; this is extensively discussed and illustrated in several papers, including those by Draisma et al. [6], Man et al. [3], and Axelsson et al. [7].

Our current study focuses on the 3D QT integral/spatial VG, leaving the QRS integral, the T integral, and the spatial QRS-T angle out of consideration.

### 1.3. Is the Ventricular Gradient Really a Gradient, and What Does It Measure?

Burger assumed that h is constant over the entire heart and has an order of magnitude of 100 mV, while τ was assumed to vary over the heart. Hence, in Burger’s theoretical framework, action potential duration differences gave the VG a value other than zero.

In 1983, Geselowitz theoretically showed that the QRST integral is determined by spatial heterogeneity in the area under the action potential, rather than by heterogeneity in action potential duration alone [8]. Hence, heterogeneity in action potential resting amplitudes, peak amplitudes, upslopes, downslopes, and action potential durations all contribute to the ventricular gradient. This somewhat more generalized concept casts the ventricular gradient into an index of heterogeneity of action potential morphology in the heart’s ventricles.

Notably, the term “ventricular gradient” is somewhat confusing because the VG is computed from the ECG as the spatial integral of the course of the heart vector. According to Burger’s relationship, this spatial QT integral equals the volume integral of action potential gradients in the heart, which is also not a gradient in the true sense of the word. However, because “ventricular gradient” has become a generally accepted term, it is not practical to change it, though it is essential to realize its physiological and mathematical background.

### 1.4. Independence of Excitation Order?

The mathematical concepts formulated by Burger [5] and Geselowitz [8] do not figure the excitation order of the heart, suggesting that the VG is independent of the ventricular activation order, entirely in agreement with Wilson’s original postulate.

The presumed independence of the ventricular activation order potentially renders the spatial VG useful in ECG diagnostics. When, in a patient, the QRST morphology changes while the VG remains the same, these changes should have been caused by differences in the depolarization order, not by changes in the intrinsic electrophysiological properties of the cardiac myocytes. However, when both the ECG and the VG change, the electrophysiological properties of the myocardium must have changed too. This property of the VG could be helpful in, for example, detecting acute ischemia in patients with a new left bundle branch block (BBB). If the acute ischemia causes the new BBB, not only the ECG but also the VG of this patient should have changed due to ischemia-based action potential changes. If the new BBB is caused by a local defect in the conduction system but unrelated to acute ischemia, the VG is expected to remain unchanged.

However, for decades the concept of VG conduction independence has met with criticism. Simonson et al. [9] did not agree with Wilson et al. [4] that the VG was “reasonably constant despite the greatly different pathways of excitation and grossly different patterns and electrical axes”. They calculated that the amount of variability in Wilson’s experiments was 25% of the mean and, therefore, should not have been interpreted as “a small variability” [4]. Angle [10] determined the VGs in the ECGs with intermittent right BBB as published by Wilson et al. [11] and White [12], and in the ECGs with intermittent left BBB as published by Segers and Boyadjian [13]. The VGs in the complexes exhibiting BBB were about half the size of those in the normally conducted complexes in right BBB, and about two thirds the size of the VGs in the normally conducted complexes in left BBB–neither supporting Wilson’s original tenet. Calculations in the above studies were done in the frontal plane. Berkun et al. [14] were the first to investigate the conduction independence of the VG in three dimensions, using a cube system of orthogonal bipolar leads for recording ECGs [15], an earlier form of uncorrected vectorcardiography. In 1966, Cosma et al. [16] stated that a three-dimensional approach to the VG requires time-integral calculations in an orthonormal vectorcardiographic lead system, because leads with unknown direction and strength can cause significant distortions in the magnitude and direction of a spatial vector. Although they do not mention Burger’s work [5], their approach fits his concept of a spatial ventricular gradient, represented by a vector with a given magnitude and spatial orientation. They compared the VG of normal sinus beats with that of premature ventricular contractions in patients with various pathology. They concluded that the VG did not remain constant when ventricular activation changed.

According to the formulae of the ventricular gradient developed by Burger [5] and Geselowitz [8], the ventricular gradient equals the volume integral of the gradients between the cellular action potential morphologies in the heart. Hence, any factor that changes action potential morphology is likely to affect the ventricular gradient. Only when all action potential morphologies in a heart change in a uniform way (e.g., an overall action potential duration increase of 10 ms) would the gradients (=local differences) in the entire heart remain unchanged, as a result, the ventricular gradient would remain the same.

After it became possible to reliably measure myocardial action potentials [17,18], the rapid development of cardiac electrophysiology has contributed much to the understanding of the processes governing excitation and recovery, thus providing greater insight into action potential distribution dynamics in the heart and the possible consequences for the ventricular gradient. In this respect, two mechanisms of particular interest are electrical restitution and electrotonic currents/potentials.

### 1.5. Electrical Restitution, S1S2 Protocol

Electrical restitution is an electrophysiological mechanism that plays a role in experiments or observations intended to provide evidence in favor or against the concept of an activation-independent VG. Experiments intending to study abnormal activation sequences often need premature stimulation to ensure pacemaker capture. The same holds for ectopic beats that might serve as an “experiment of nature” to alter the cardiac activation order: in most cases, ectopic foci must fire prematurely to become manifest ectopic beats. Hence, an abnormal ventricular activation sequence generally requires premature stimulation or a premature extrasystole. However, prematurity alters the action potential morphology.

The electrophysiological effects of premature stimulation of the heart are commonly described by the electrical restitution curve, originally defined by Bass in 1975 [19]. This curve represents the recovery time course of action potential duration (APD) as a function of the diastolic interval (DI) between a steady-state APD at a given stimulation cycle length (CL) and the response to an extrastimulus [20]. In experimental and clinical electrophysiology, this stimulation protocol is denoted as the S1S2 restitution protocol (S1 representing the steady-state stimuli, S2 representing the extrastimulus).

VG changes due to S1S2 restitution are caused by restitution heterogeneity in the heart: regional differences in the APD change in response to the premature stimulus. The study by Srinivasan and colleagues [21], set up to measure dynamics in repolarization dispersion, yields valuable insights in this respect. They measured, in structurally normal hearts, activation-recovery intervals (ARI) as APD surrogates in a setting with (1) a right-ventricular decapolar electrode for recording in an apicobasal orientation in the right ventricle, (2) a decapolar catheter on the lateral wall of the left-ventricular endocardium for recording in an apicobasal orientation in the left ventricle, and (3) a decapolar catheter on the epicardium of the left ventricle via the lateral cardiac vein via the coronary sinus for recording transmurally across the left-ventricular wall. The authors observed differences between the apical and basal restitution curves of the right and left ventricles, and between the epicardial and endocardial restitution curves in the left-ventricular free wall. In addition, the restitution curves differed depending on the pacing site (either right-ventricular endocardial, left-ventricular endocardial, or left-ventricular epicardial); see their Figure 5.

When the restitution curves measured at various sites in the heart run parallel, changes in action potential duration due to a premature stimulus will be the same, and the VG will not be affected. When the restitution curves are not parallel, the regional action potential changes are not the same, which implies restitution heterogeneity and, as a consequence, changes in the VG.

From the data published by Srinivasan and colleagues, it is difficult to predict what will happen in a specific case, characterized by a certain degree of prematurity and a specific stimulus site or the site of origin of a ventricular extrasystole. Restitution curves were only measured, and pacing was only performed at a limited number of sites. However, a general conclusion can be that there may be situations with given prematurity and a stimulus site in which all APDs exhibit a similar change and the VG will not or barely change, while there may also be situations in which this is not the case. Particularly in situations involving stimuli with higher prematurity (diastolic interval < 300 ms) the curves are sometimes less parallel, while left-ventricular endocardial pacing seems to induce the most heterogeneous restitution curves.

### 1.6. Electrical Restitution, Change in Stimulation CL

Another more complex manifestation of electrical restitution is the dependence of APD on stimulation cycle length. Franz et al. [22] showed that the transient from a steady-state APD at a given stimulation cycle length (CL) towards a new steady-state APD after a step change in stimulation CL takes several minutes, or several hundreds of beats. More dynamic CL changes (e.g., a gradual decrease followed by a gradual increase) that are too fast for steady-state APD adaptation can cause hysteresis in the APD–CL relationship [23,24] and, consequently, in the ECG (e.g., during the exercise and recovery phase of an exercise test [25]).

Laboratory experiments in preparations from dog hearts have shown that, after adaptation to a step decrease in CL, epicardial action potentials shorten more than endocardial action potentials [26]. This heterogeneous restitution in the setting of a rhythm change supports the idea that the VG is sensitive to changes in rhythm. Indeed, our transversal study of young adults [27] has shown that the VG magnitude decreases with increasing heart rate. Additionally, a dynamic study of the VG during exercise and subsequent recovery in healthy male subjects showed (with hysteresis) a decrease in VG magnitude with increasing heart rate. Axelsson and colleagues [28] showed that VG magnitude decreased after adaptation to 120 bpm supraventricular pacing or ventricular pacing in patients with permanent pacemakers due to sick sinus syndrome or reflex syncope (coronary heart disease, diabetes, or bundle branch block excluded). These observations have led us to conclude that studies regarding the dependence of VG on conduction cannot be done in combination with a heart rate change, as it would modify or mask the VG’s response to a change in cardiac activation order.

### 1.7. Electrotonic Effects

Cardiac cells are electrically connected; electrotonic currents flow through gap junctions between cells as a consequence of differences in intracellular potential [29]. This implies that neighboring cells influence each other electrically, during activation/depolarization, in the course of their action potential, and during recovery/repolarization. The region of influence is characterized by the space constant; usually, this constant has a magnitude of some millimeters. Due to electrotonic interactions, the action potential of myocardial cells partly represents the characteristics of their environment. Thus, the action potential of a cell can be different depending on the electrophysiological properties of the myocytes in the direction from which the depolarization and repolarization wavefronts arrive.

To investigate the presumed independence of the VG on the ventricular activation order, Lux and colleagues made body surface potential maps in dogs with various pacing modalities, measured QRST integrals at all 192 electrode sites (lead voltage = local electrode potential versus the Wilson central terminal potential) and constructed QRST iso-area maps [30,31]. They concluded that “QRST iso-area maps were shown to be independent of ventricular activation sequence with the exception of features which are explicable on the basis of electrotonic interaction during ventricular repolarization” [32] and “it appears that the influence of activation order on recovery properties should be considered in evaluating the significance of a given value of the ventricular gradient” [33].

In our view, these observations, in which electrotonic effects are used to explain the possible dependence of the VG on ventricular activation order, are not essentially different from the S1S2 restitution curve measurements by Srinivasan and colleagues [21].

In intact hearts, S1S2 restitution curves cannot be measured for isolated areas: they necessarily include the electrotonic effects of the activation-recovery pattern associated with the site of stimulation/origin of the extrasystole.

### 1.8. Purpose of Our Study

In conclusion, we consider the S1S2 restitution curves as measured by Srinivasan and colleagues [21] as a valid set of observations providing an experimental background for ECG-based studies in which extrasystoles are used to test the concept of conduction independence of the ventricular gradient. We designed an “experiment of nature”, in which the VG of a spontaneous supraventricular or ventricular extrasystole occurring in a standard diagnostic 12-lead ECG is compared with the VG of the sinus beats. Our study remains within the realm of vectorcardiography and can best be compared with the study by Cosma and colleagues [16]. There are, however, three essential differences:

- As well as including ventricular extrasystoles as per Cosma et al.’s study, we included patients with supraventricular extrasystoles;
- Instead of comparing the ventricular gradient vector magnitude of the sinus beat and extrasystole, we compared the ventricular gradient vectors, thus considering any differences in magnitude, direction, and combinations as a change;
- We included the prematurity of the extrasystoles in our analysis.

## 2. Materials and Methods

### 2.1. Study Design

Anonymous, retrospective, observational.

### 2.2. ECG Inclusion

ECGs were drawn from the ECG database of the Leiden University Medical Center (LUMC) in which, since 1986, the ECGs recorded in the LUMC have been stored. Within an arbitrarily chosen 10-month period, we collected all technically sound digital standard 10-s 12-lead ECGs of patients > 18 years old that satisfied the following criteria:

- The ECG shows a normal sinus rhythm;
- The ECG shows at least one spontaneous supraventricular or ventricular ectopic beat;
- The predominant supraventricular beat has a well-identifiable J point, allowing for reliable determination of the QRS duration;
- The predominant beat does not fulfill the criteria for a strict left bundle branch block (LBBB) configuration [34].

Of note: the wording “predominant beat” refers to the most frequently occurring beat in a particular ECG recording; it may have a normal or pathological QRST waveshape, depending on the patient.

### 2.3. ECG Exclusion

For this study, we applied an ECG selection strategy to ascertain several conditions that were technically needed for reliable ECG analysis. ECGs were excluded if they did not comply with one or more of the following conditions:

- The ECG facilitated a reliable baseline assessment throughout the entire recording, including the ectopic beat of interest;
- A context of at least four predominant supraventricular beats preceding the ectopic beat, as well as at least one predominant supraventricular beat immediately following the ectopic beat with a compensatory pause, was required;
- To ascertain stable cellular electrophysiology, the minimum four predominant supraventricular beats preceding the ectopic beat had to pass a check for rhythmic regularity: ECGs with intervals between predominant beats that differed more than 10% from the average interval were excluded;
- In ECGs that contained multiple ectopic beats that satisfied the selection criteria, only the first ectopic beat was analyzed;
- In patients with multiple ECG recordings that satisfied the selection criteria, only the first recording was analyzed (to prevent double inclusion of patients).

### 2.4. Computerized ECG Analysis

The ECGs were analyzed and generally characterized by the Glasgow Royal Infirmary (GRI) software [35], thus labeling each ECG as normal or abnormal, based not only on the properties of the predominant beat but also on rhythm and conduction disorders as detected in the ECG. We then summarized the GRI-detected abnormalities into the following categories: long QT, axis deviations, BBBs, intraventricular conduction disorders, hypertrophy, old infarctions, pericarditis, ST abnormalities, and low QRS voltage.

The ECGs were additionally analyzed by the Leiden ECG Analysis and Decomposition Software (LEADS) [36]. Briefly, LEADS combines automated analysis and human interaction to identify the heartbeats of interest in an ECG recording, as well as verifying and, when necessary, adjusting the essential landmarks in time (onset of the QRS complex and end of the T wave, together defining the interval for the computation of the VG). Initially, LEADS computes a vectorcardiogram (VCG) by multiplying the ECG by the Kors transformation matrix [37]. Heartbeats are then detected in the VCG-derived spatial velocity signal [36]. Next, ectopic beats are excluded, thus focusing on the analysis of the predominant QRST complex. Before averaging the predominant beats, the baseline is corrected by cubic spline interpolation through the PQ segments immediately preceding the predominant QRS complexes.

For the purpose of this study, ectopic beats also had to be analyzed. Given ectopic beats usually miss an isoelectric segment preceding their onset, they do not contribute to baseline estimation. To facilitate the analysis of a separate ectopic beat, a special experimental version of the LEADS program was developed, in which the ectopic beats were baseline-corrected, with the baseline as calculated from the surrounding predominant beats in the ECG recording.

### 2.5. Further Calculations

From the variables produced by LEADS, we calculated the prematurity of the ectopic beat (the ratio of the coupling interval of the ectopic beat and the mean interval between consecutive predominant beats). From the VG vectors of the predominant and ectopic beats, $\vec{VG_{p}}$ and $\vec{VG_{e}}$, we computed three measures of difference:

1. The magnitude of the difference vector between the VGs of the ectopic and predominant beats: $\left|\vec{\Delta VG_{ep}}\right|$;
2. The magnitude of the difference between the VG magnitudes of the ectopic and predominant beats: $\left|\vec{\left|VG_{e}\right|}-\vec{\left|VG_{p}\right|}\right|$;
3. The angle α between the VGs of these two beats.

**Figure 1:**
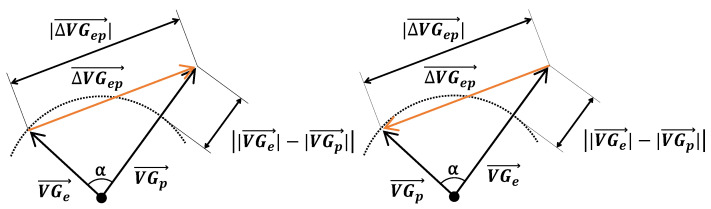
Relationship between $\vec{VG_{e}}$ (the ventricular gradient of the ectopic beat) and $\vec{VG_{p}}$ (the ventricular gradient of the predominant beat).$\;\left|\vec{VG_{e}}\right|$ and $\left|\vec{VG_{p}}\right|$ are the magnitudes of $\vec{VG_{e}}$ and $\vec{VG_{p}}$, respectively. $\left|\vec{\left|VG_{e}\right|}-\vec{\left|VG_{p}\right|}\right|$ is the magnitude of the difference between the magnitudes of $\vec{VG_{e}}$ and $\vec{VG_{p}}$, irrespective of which is the largest and which is the smallest.$\;\left|\vec{\Delta VG_{ep}}\right|$ is the magnitude of the difference vector between $\vec{VG_{e}}$ and $\vec{VG_{p}}$. This difference vector magnitude depends on $\left|\vec{\left|VG_{e}\right|}-\vec{\left|VG_{p}\right|}\right|$ and on the angle α between the two ventricular gradient vectors. If $\left|\vec{\Delta VG_{ep}}\right|$ equals $\left|\vec{\left|VG_{e}\right|}-\vec{\left|VG_{p}\right|}\right|$, the ventricular gradient vectors of the predominant and ectopic beat assume the same direction. A difference in spatial orientation of $\vec{VG_{e}}$ and $\vec{VG_{p}}$ increases the magnitude of the difference vector $\left|\vec{\Delta VG_{ep}}\right|$, which is then larger than $\left|\vec{\left|VG_{e}\right|}-\vec{\left|VG_{p}\right|}\right|$. The larger the angle between $\vec{VG_{e}}$ and $\vec{VG_{p}}$, the larger the difference between $\left|\vec{\Delta VG_{ep}}\right|$ and $\left|\vec{\left|VG_{e}\right|}-\vec{\left|VG_{p}\right|}\right|$. The figure illustrates this, including the invariance for the difference in magnitudes of $\left|\vec{VG_{e}}\right|$ and $\left|\vec{VG_{p}}\right|$: in the left panel $\left|\vec{VG_{e}}\right|$ is smaller than $\left|\vec{VG_{p}}\right|$, while in the right panel $\left|\vec{VG_{e}}\right|$ is larger than $\left|\vec{VG_{p}}\right|$.

Figure 1 is a pictorial representation of the relationship between these measurements. It shows how the magnitude of the difference vector between the VGs of the ectopic and predominant beats combines differences in the VG magnitudes of the ectopic and predominant beats, and differences in their spatial orientations. Our study primarily focuses on the difference between $\vec{VG_{e}}$ and $\vec{VG_{p}}$, irrespective of whether this is caused by a difference in magnitude, a difference in spatial orientation, or a combination thereof.

### 2.6. Ectopic Beat Categorization

We divided the ectopic beats into three categories: supposed supraventricular origin and normal conduction (i.e., similar conduction as the predominant beat; SN), supposed supraventricular origin and aberrant conduction (i.e., different conduction compared to that of the predominant beat; SA), and supposed ventricular origin (V).

**Figure 2:**
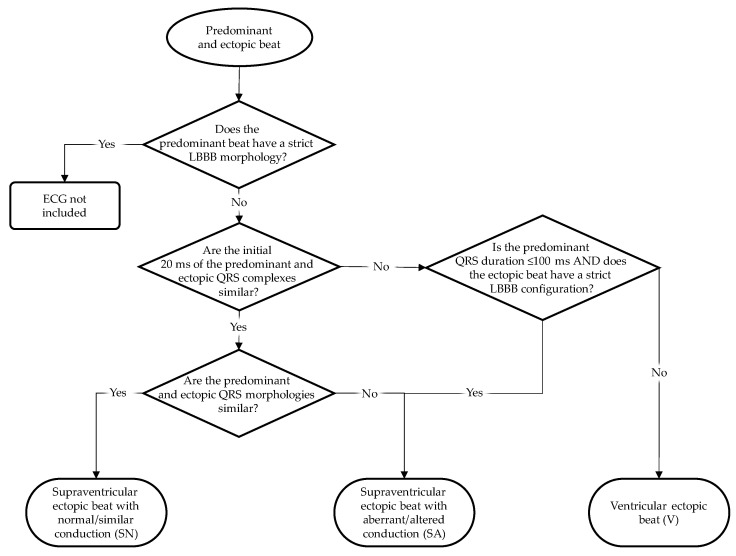
Flowchart for ectopic beat categorization into beats of supposed supraventricular origin with and without aberrant conduction and beats of supposed ventricular origin. See text.

Figure 2 shows the categorization scheme of the ectopic beats. To discriminate supraventricular from ventricular ectopic beats, we compared the initial 20 ms of the ectopic beat and the predominant beat of the same patient, as suggested by several studies [38,39,40,41]. This discrimination method relies on the presupposition that the initial ventricular activation of a supraventricularly initiated QRS complex starts by left-ventricular septal wall activation from the left bundle branch. Similar initial 20 ms of the predominant and ectopic QRS complexes would then imply that the ectopic QRS complex is of supraventricular origin. Consequently, patients with a strict LBBB QRS configuration in their predominant beat were excluded from our study. In the case of an incidental premature beat with strict LBBB configuration, we consider it as a supraventricular aberrantly conducted ectopic beat. This is the only option for ectopic beats with differing initial 20 ms compared to the predominant beat to be classified as supraventricular (aberrantly conducted because of the LBBB).

Comparisons of the initial 20 ms in the predominant and ectopic beats have been made by careful inspection of the ECGs in a multi-channel display of all ECG leads over 10 s by four of the authors (ACM, CAS, MD, RMS) jointly, thus removing subjectivity as much as possible. The decision about the similarity of the initial 20 ms of the ectopic and predominant QRS complex was taken on the basis of similar polarity and rate of rise/fall [38,39,40,41]. Once ectopic QRS complexes had been labeled supraventricular, their conduction pattern was classified as normal or aberrant, depending on the comparability of the ectopic and predominant QRST configurations.

The separation into ectopic beats of supposed supraventricular origin with and without aberrant conduction, and ectopic beats of supposed ventricular origin, was made to contrast the influence of solely restitution on the VG (by comparing ectopic beats of supposed supraventricular origin and sinus beats) and the combined influences of restitution and activation order differences on the VG (by comparing sinus beats on one hand and aberrantly conducted ectopic beats of supraventricular origin and ectopic beats of ventricular origin on the other hand).

### 2.7. Statistics

To compare $\vec{VG_{p}}$ and $\vec{VG_{e}}$, we calculated the mean and standard deviations of the $\vec{VG_{p}}$ and $\vec{VG_{e}}$ magnitudes, testing the difference of the means by a two-sided paired T test. Additionally, we computed the mean and standard deviation of the VG difference vector magnitude, $\left|\vec{\Delta VG_{ep}}\right|$. Values were computed for all patients and for the subgroups of patients with ectopic beats in the SN, SA, and V categories. Finally, we statistically compared the values of these subgroups by two-sided unpaired T tests and performed linear regressions.

## 3. Results

### 3.1. Study Group

**Figure 3:**
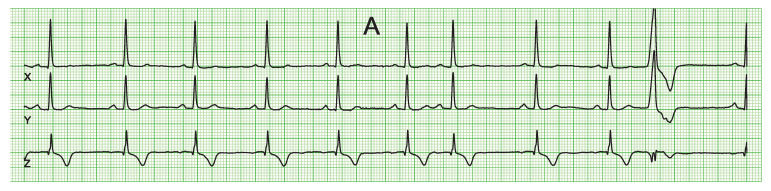

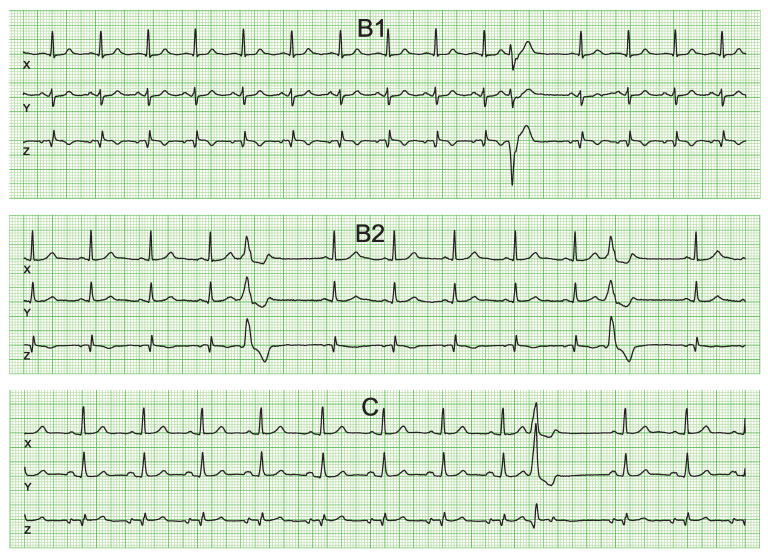
Examples of VCGs of subjects with a supraventricular ectopic beat with normal conduction (**panel A**, beat seven was analyzed), a supraventricular ectopic beat with similar initial 20 ms and aberrant conduction (**panel B1**), a supraventricular ectopic beat with strict LBBB configuration, and thus also aberrant conduction (**panel B2**, beat five was analyzed), and a ventricular ectopic beat (**panel C**), respectively.

After querying the ECG database, 375 ECGs matched the inclusion criteria, of which 257 ECGs were excluded due to the reasons listed in Table 1. This left 118 ECGs of 118 unique patients as study data. Figure 3 shows VCG examples of a subject with a premature supraventricular ectopic beat with normal conduction (panel A), a premature supraventricular ectopic beat with aberrant conduction—with similar initial 20 ms (panel B-1) or with complete LBBB (panel B-2) —and with a ventricular ectopic beat (panel C).

Table 2 shows the anthropomorphic characteristics of the study group. Two-thirds of the patients were male. The average BMI was 27.7 kg/m^2^; 72.8% of patients had a BMI > 25.0 kg/m^2^; 25.4% of patients were obese (BMI > 30.0 kg/m^2^). Table 3 shows the cardiovascular diagnoses in the study group; an average of 2.2 cardiovascular conditions were present per patient. All included ECGs had been previously recorded in the course of routine clinical procedures in a cardiology department (these reasons did not play a role in the selection of our studied ECGs). Table 4 shows a breakdown of the clinical reasons for the recording of the ECGs in our study group. In most patients (82.2%), the ECGs were recorded during an outpatient-clinic visit for periodic screening or diagnostic workup. In a minority of our study group, the ECGs were recorded in the setting of an elective intervention (e.g., ICD implantation) or an acute event (e.g., admission after out-of-hospital cardiac arrest). The mean ± SD heart rate was 74.9 ± 10.1 bpm and the range 57.2–98.4 bpm. Table 5 gives a breakdown of the QRS durations of the predominant beats for patients with normally conducted supraventricular ectopic beats, aberrantly conducted supraventricular ectopic beats, and ventricular ectopic beats. Not unexpectedly, the mean predominant QRS duration of patients with ventricular ectopic beats was significantly larger than that of the patients with normally conducted supraventricular ectopic beats.

### 3.2. GRI-Based ECG Interpretation

Table 6 shows the ECG qualification by GRI software. The majority of ECGs (72.0%) were interpreted as abnormal or borderline abnormal. Table 7 shows the detailed GRI interpretation. ST abnormalities were the most common. Among the seven ECGs that were recorded in acute conditions (see Table 4), three patients were diagnosed with ST abnormalities. From this, it follows that 52 − 3 = 49 patients had ST abnormalities in non-acute conditions, possibly related to conduction disorders, old infarctions, and hypertrophy, all present among the abnormalities as listed in Table 7.

### 3.3. Relationship between the Magnitudes of the Ventricular Gradients of Predominant and Ectopic Beats

**Figure 4:**
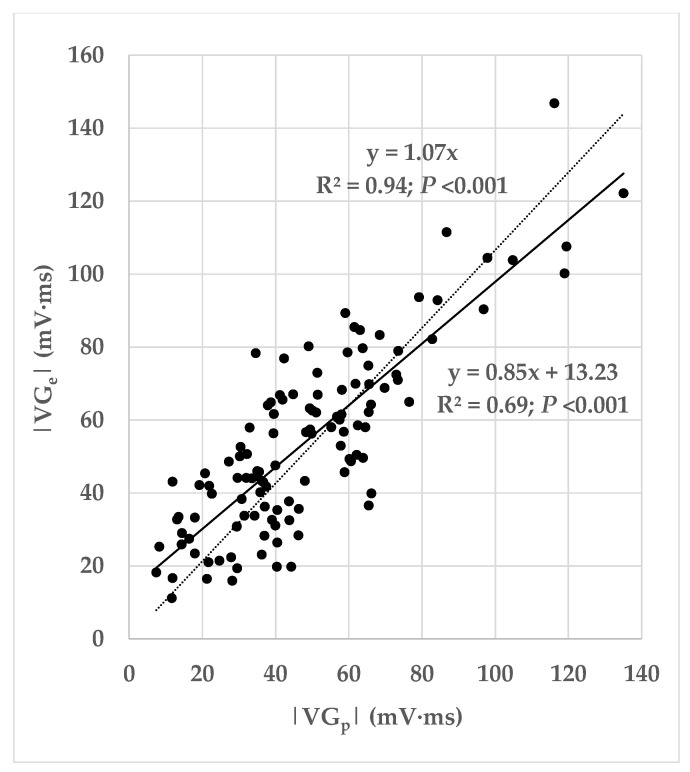
Magnitudes of the ectopic ventricular gradient ($\left|\vec{VG_{e}}\right|$) versus the ventricular gradient of the predominant beat ($\left|\vec{VG_{p}}\right|$). The scatterplot displays the data from all patients (N = 118). The solid line shows the linear regression with constant (intercept = 13.23) and the dotted line shows the linear regression forced through the origin (intercept = 0). The correlation coefficients of both regressions differ statistically significantly from zero (*p* < 0.001).

Figure 4 shows all measurements as a scatterplot of the ventricular gradient magnitudes of the ectopic beats versus those of the predominant beats. Both straightforward linear regression and linear regression forced through the origin yielded significant correlation coefficients (*p* < 0.001), slopes close to 1, and, for the linear regression forced through the origin, an R-squared value that explains nearly all variance in the dependent variable.

### 3.4. Angles between the Ventricular Gradient Vectors of the Predominant and Ectopic Beats

**Figure 5:**
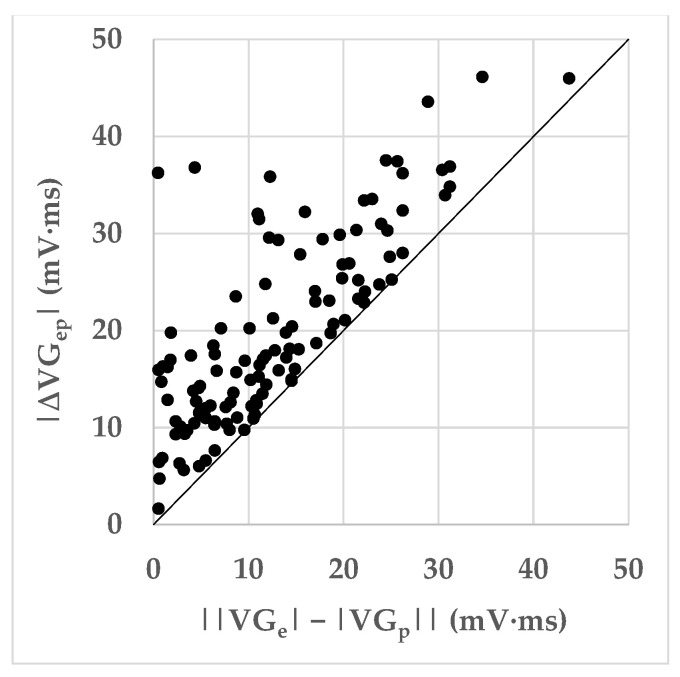
Magnitude of the ventricular gradient difference vector ($\left|\vec{\Delta VG_{ep}}\right|$) versus the magnitude of the difference between the ventricular gradient magnitudes ($\left|\vec{\left|VG_{e}\right|}-\vec{\left|VG_{p}\right|}\right|$). When $\vec{VG_{e}}$ and $\vec{VG_{p}}$ have the same spatial orientation, the magnitude of the difference vector equals the difference of the vector magnitudes. This situation is represented by the 45-degree line. When $\vec{VG_{e}}$ and $\vec{VG_{p}}$ have different spatial orientations, the magnitude of the difference vector is larger than the difference of the vector magnitudes (data points above the 45-degree line). The scatterplot shows that not only the magnitudes of the $\vec{VG_{e}}$ and $\vec{VG_{p}}$ differ, but that the spatial orientations differ as well. See also Figure 2.

Figure 5 shows all measurements as a scatterplot of the magnitude of the ventricular gradient difference vector versus the magnitude of the difference between the ventricular gradient magnitudes. As a consequence of the vectorial calculus rules, and as shown by the figure, the magnitudes of the difference vector are never smaller than the difference between the magnitudes of the ventricular gradients of the predominant and ectopic beats. Any difference implies that the ventricular gradient vectors of the predominant and ectopic beats are not pointing in the same directions in space (see Figure 1).

**Figure 6:**
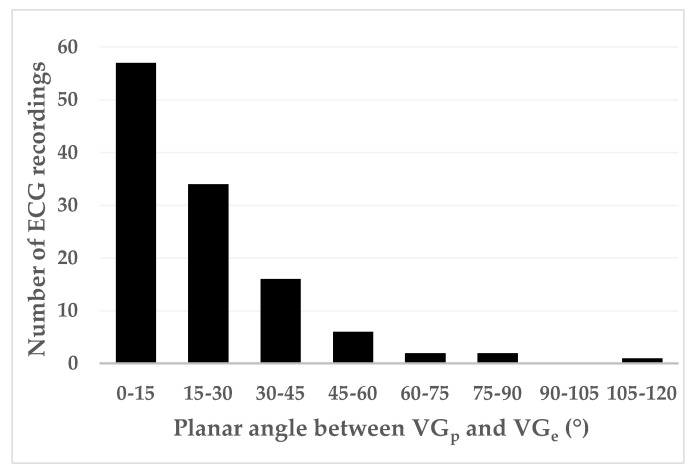
Distribution of the planar angle α (see Figure 2) between the ventricular gradient vectors of the ectopic beat ($\vec{VG_{e}}$) and of the predominant beat ($\vec{VG_{p}}$).

Figure 6 shows the distribution of the planar angles between the VG vectors of the predominant and ectopic beats. The mean planar angle between the VG vectors of the predominant and ectopic beats is 21.0°. If we arbitrarily separate smaller and larger angles by a threshold of 30°, a majority (77.1%) of planar angles appear to be relatively small. However, in a considerable number of cases (22.9%), the planar angle between the VG vectors of the predominant and ectopic beats is relatively large. The mean ± SD planar angles between the VG vectors of the predominant and ectopic beats were 24.2° ± 39.8°, 23.8° ± 22.6°, and 20.2° ± 15.7° for patients with normally conducted supraventricular ectopic beats (SN), aberrantly conducted supraventricular ectopic beats (SA), and ventricular ectopic beats (V), respectively. Mutual comparison of these mean planar angles did not yield statistically significant differences.

### 3.5. Influences of the Presumed Origin, Intraventricular Conduction, and Prematurity of the Ectopic Beat on the Magnitude of the Ventricular Gradient Difference Vector

Table 8 shows that the mean magnitude of the ectopic VG vector was significantly larger than the mean magnitude of the predominant VG vector in patients with supraventricular ectopic beats with aberrant conduction (*p* = 0.04) and in patients with ventricular ectopic beats (*p* < 0.001). No other statistically significant differences emerged, although the impression arises that the VG magnitudes of the predominant beats of patients with aberrantly conducted supraventricular ectopic beats and of patients with ventricular ectopic beats are smaller than those of patients with normally conducted supraventricular ectopic beats. The limited number of patients with normally conducted supraventricular ectopic beats (N = 6) and the unpaired statistical testing precluded further conclusions regarding this observation.

**Figure 7:**
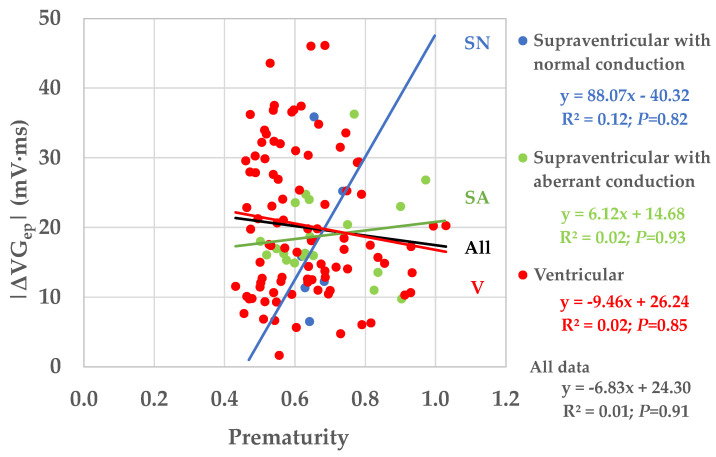
Magnitudes of the ventricular gradient difference vectors as a function of prematurity for patients with normally conducted supraventricular ectopic beats (SN; N = 6), aberrantly conducted supraventricular ectopic beats (SA; N = 20), and ventricular ectopic beats (V; N = 92). Linear regressions were computed from the VG difference vectors of these three subgroups and of the entire study group (All; N = 118) on prematurity. All correlation coefficients were very low and none of the regression lines had a slope that was statistically significantly different from zero.

Figure 7 shows the magnitudes of the ventricular gradient difference vectors as a function of prematurity for patients with normally conducted supraventricular ectopic beats (SN; N = 6), aberrantly conducted supraventricular ectopic beats (SA; N = 20), and ventricular ectopic beats (V; N = 92). Four linear regressions were computed from the VG difference vectors of these three groups and of the entire study group (All; N = 118). All correlation coefficients were very low and none of the regression lines had a slope that was statistically significantly different from zero. It appears from the data, though, that the larger values of the VG difference vectors occur at prematurity values < 0.8.

## 4. Discussion

We investigated the dependence of the VG on activation order and restitution by analyzing standard diagnostic ECGs with a spontaneous ectopic beat, measuring intra-individual differences between the VGs of predominant and ectopic beats in 118 patients (Table 2) with various cardiovascular pathology (Table 3) and with, for the greater part, abnormal ECGs (Table 6). Prematurity varied widely (0.43–1.03; Figure 7), thus covering almost the entire electrophysiologically possible range.

Ectopic VG vector magnitudes were significantly larger than predominant VG magnitudes for SA and V, but not for SN ectopic beats (Table 8). VG difference vectors were larger than the differences between the predominant and ectopic VG magnitudes, signaling predominant-ectopic differences in spatial VG orientation (Table 8 and Figure 5). Magnitudes of the VG difference vectors were uncorrelated with the prematurity of the ectopic beats (Figure 7), but the larger values of the VG difference vectors occur at prematurities below 0.8.

If we would interpret the VG magnitude as measuring the heterogeneity of ventricular action potential (AP) morphologies, the conclusion would be that ectopic beats bring about an increase in AP heterogeneity. We must realize, however, that the VG is—like many other ECG phenomena—subject to cancellation. Hence, when including cancellation in the VG interpretation, it may be more correct to interpret the VG as the net resulting AP heterogeneity. Depending on the net resulting AP heterogeneity of an individual patient’s predominant beat (represented by $\vec{VG_{p}}$), and depending on the AP changes in an ectopic beat of this patient (brought about by restitution and differences in activation order), the net resulting AP heterogeneity in the ectopic beat (represented by $\vec{VG_{e}}$) may either be larger or smaller than the net AP heterogeneity of the predominant beat (see Figure 4). In our database, the averaged ectopic VG magnitudes are larger than the averaged VG magnitudes of the predominant beats (Table 8). Due to the cancellation effect involved in VG measurement, the impact of this finding remains uncertain: several patients appear to have smaller $\left|\vec{VG_{e}}\right|$ than $\left|\vec{VG_{p}}\right|$ values (see Figure 4).

Figure 6 shows that differences in spatial orientation of the predominant and ectopic VG vectors are common; this contributes to the VG difference vector (see Figure 1). Figure 7 shows that restitution effects are not systematically occurring: the linear correlation between the VG difference vector magnitude and prematurity are very low and not statistically significantly different from zero. The scatterplot suggests, however, that larger VG difference vector magnitudes do not occur with ectopic beats with modest (>0.8) prematurity.

The durations of the predominant QRS complexes varied widely (Table 5). The mean QRS durations of the patients with ventricular ectopic beats (108.8 ± 22.4 ms) were significantly larger than those of the patients with supraventricular ectopic beats with normal conduction (90.0 ± 18.0 ms). This may indicate a higher prevalence of intraventricular conduction abnormalities in these patients.

### 4.1. Comparison with Previous Studies

Lux et al. [30] made QRST-area maps in dogs during pacing in the right atrium, right atrium plus right ventricle, right atrium plus left ventricle, and in the right atrium plus simultaneously in the left and right ventricles. Pacing stimulation rates were just fast enough to achieve capture; hence, comparisons of these QRST-area maps were done at nearly the same basic cycle length.

Body-surface QRST-area maps are strongly related to the VG. When QRST-area maps do not change under various experimental conditions, this implies that VG remains constant under those conditions. Given that, in the experiments by Lux and colleagues, the QRST-area maps remained strikingly similar under these different pacing modalities, the authors favored the conclusion that VG is independent of the ventricular activation order.

Due to all experiments of this group described in Lux et al. [30] including a form of atrial pacing (even when ventricular pacing was performed, this was always combined with atrial pacing), their measurements never showed purely ventricular ectopic activation. This, and the limited range of prematurity (prematurity was restricted to what was needed to overpace the sinus node), hampers extrapolation of their data to premature ventricular contractions, where the degree of prematurity and the ventricular activation sequence differences are more prominent. This may have led to the rather optimistic conclusions regarding VG conduction independence in this study.

Earlier, however, the same group published results of similar experiments [30] from which they concluded that “QRST iso-area maps were shown to be independent of ventricular activation sequence with the exception of features which are explicable on the basis of electrotonic interaction during ventricular repolarization” [31]. The 1978 publication [30] gives two striking examples. Their Figure 1 visually compares the QRST-area maps obtained by stimulating a dog heart in the right atrium and in the left ventricle, both at a cycle length of around 400 ms. The QRST-integral maps resemble each other strikingly. Their Figure 2 visually compares, in the same experiment, the QRST-area maps obtained by slightly and strongly premature right-ventricular stimulation. The QRS-T integral map obtained by slightly premature right-ventricular stimulation closely resembles the QRS-T integral maps in Figure 1, while the QRS-T integral map obtained with strongly premature right-ventricular stimulation is considerably different. Given these are just single observations, the findings must be interpreted with care but they suggest that, in this experiment, prematurity is more important for differences in QRST-integral maps (and, hence, in VG) than the stimulation site/activation order.

Berkun et al. [14] studied patients with intermittent pre-excitation, with intermittent left bundle branch block, and with premature ventricular contractions. The differences between the VGs of the normal sinus beats and the VGs of the premature ventricular contractions were strikingly larger than the differences between the VGs of the normal sinus beats and the VGs of the sinus beats with pre-excitation or with left bundle branch block. Comparison of our study results and those of Berkun and colleagues is not straightforwardly possible. In the first place, the number of patients in the Berkun and colleagues study is very limited (four pre-excitation, six left bundle branch block, eight premature ventricular contractions), preventing statistical analysis. Additionally, the beats with pre-excitation or with left bundle branch block are essentially sinus beats, hence not premature. The beats with left bundle branch block represent only a limited variation in the ventricular activation order when compared to ventricular ectopic beats, of which the ectopic pacemaker can virtually be localized everywhere in the ventricles. Our study used a much larger group to compare VG changes in supraventricular and ventricular ectopic beats, both having abnormal timing, but differing in activation order. Finally, as already mentioned in the introduction, Berkun and colleagues were the first to investigate the conduction independence of the VG in three dimensions, but they used a cube system of orthogonal bipolar leads for ECG recording [15]. This is an earlier form of uncorrected vectorcardiography: the lead vector directions may be quite correct, but the sensitivities of the leads may differ. This potentially gives inaccuracies in the size and direction of the heart vector.

Cosma et al. [16] compared the magnitudes of the spatial VG of normal sinus beats with the magnitudes of the spatial VG of premature ventricular contractions in VCG recordings of patients with various pathologies. They concluded that “the VG […] does not remain constant when the pathway of ventricular activation changes”. Given the prematurity of the extrasystoles was not considered in their study, the contribution of prematurity to their results cannot be assessed.

Our study suggests that the contribution of prematurity to the VG difference vector magnitude is complex: on one hand, it seems that a minimal amount of prematurity—about 0.80—is required to generate larger values of the VG difference vector magnitude; on the other hand, below the value of 0.80 there is no clear relationship between the amount of prematurity and the VG difference vector magnitude, therefore either small or large difference values may occur at the same amount of prematurity.

### 4.2. Interpretation of Our Results

The VG changes induced by an extrasystole can be understood from the electrical restitution properties of the entire heart, as per the restitution curves of 10 patients with structurally normal hearts published by Srinivasan and colleagues [21]; see their Figure 5. Given the restitution curves were made in intact hearts, they necessarily include electrotonic effects. Notably, the restitution curves in their Figure 5 are locally weighted scatterplot smoothing (LOWESS) curves of all studied patients. As a consequence of the LOWESS procedure, the restitution curves look very smooth. Separate restitution curves will undoubtedly be more complex, and with abnormal hearts and with more measurement and stimulation sites, more capricious restitution behavior is likely to be observed. However, in the measurements presented in their study, we can see that the restitution curves in different parts of the heart do not always run in parallel and certainly behave differently with substantial prematurity of the ectopic stimulation. Given the above-described restitution curve properties, our results (varying amounts of sometimes large VG difference vector magnitudes for extrasystole prematurities < 0.80, see Figure 7) are understandable. Our findings also correspond with those of Cosma et al. [16]. In their Table 3, they give the VG magnitudes of the sinus beats and the extrasystoles of 10 randomly selected subjects. We see a large variability of VG magnitude differences, the smallest being 3.5 mV∙ms and the largest 74.6 mV∙ms (as we compute the VG difference vector magnitude, our values might even have been larger if the VG of the extrasystole had a different spatial orientation than the VG of the sinus beats).

Our results show that prematurity is always an issue when comparing the VG of sinus beats and extrasystoles, no matter whether they are normally or abnormally conducted. For example, we saw a few cases with large VG difference vector magnitudes for premature supraventricular extrasystoles with normal conduction (see Figure 7). Therefore, we may conclude that individual VG comparisons of sinus beats and extrasystoles (or paced beats) require that the extrasystole or paced beat is not or almost not premature. We have too little data for a meaningful comparison of the VG of sinus beats and late (i.e., non-premature) abnormally conducted extrasystoles.

The study by Lux et al. [30], in which different conduction patterns were generated by pacing, made comparisons of the QRST-integral body surface maps at almost the same heart rate. The body surface maps appeared to be almost independent of the stimulation sites. An earlier experiment with the same group [31] suggests that QRST-integral body surface maps are sensitive to prematurity rather than to a different conduction pattern.

### 4.3. Methodological Considerations

Validation of the concept of an activation-independent VG is, in several respects, a methodological challenge. When comparing, within subjects, the VG of a representative QRST complex (e.g., the QRST complex associated with sinus rhythm) with the VG of a QRST complex with a different ventricular activation order, we should take into account that several physiological mechanisms can alter the APD distribution in the heart, and thus affect the QRST integral in Burger’s formula. Only when the APDs remain the same, or lengthen or shorten homogeneously, is the VG unaffected because the spatial differential (nabla) in the Burger formula remains unaffected. Examples can be seen in Figure 2 of Draisma et al. [6]. The ECGs in this figure were obtained by simulations in which the action potential morphology was parametrized and could not dynamically change. However, in real life this is seldom the case; several electrophysiological effects play a role [42] that impact the experimental setup of VG validation studies.

#### 4.3.1. Restitution

Our current study suggests that restitution effects cause heterogeneous APD changes throughout the heart, thus affecting the cell-to-cell APD gradients (the nabla in Burger’s formula). This would mean that intra-individual VG comparisons should be made either at equal heart rates (in the case that two separate ECG recordings are compared, of which, e.g., one ECG has regular intraventricular conduction and the other shows a situation of intraventricular conduction block or accelerated idioventricular rhythm). Alternatively, comparisons should be made between the VGs derived from the predominant QRST complex and derived from a non-premature extrasystole or from an incidental QRST complex with an intraventricular block.

#### 4.3.2. Autonomic Nervous System

Because the effects of autonomic nervous system (ANS) activity are non-homogenously distributed over the heart, e.g., with different APD responses in the epicardium and endocardium, ANS activity should preferably be the same during the time of occurrence of the reference QRST pattern and that of a deviating QRST pattern. Constant ANS activity is likely the case when a single extrasystole interrupts a regular supraventricular rhythm. The extrasystole will usually have a lower stroke volume, hence the systolic pressure of the pulse wave in the aorta and carotid arteries will be lower (or even absent) for the extrasystolic beat. This has, via the arterial baroreflex, consequences for the parasympathetic and sympathetic ANS outflow (with an extrasystolic beat, there will be a decrease in parasympathetic outflow and an increase in sympathetic outflow). This causes an increase in heart rate and in cardiac contractility. However, such reflex responses occur only after a delay of one or more seconds, not during the extrasystole itself. Hence, the VG of a single non-premature extrasystole or the VG of an interrupting sinus beat with an intermittent bundle branch block can be compared with the VG of the predominant beat.

#### 4.3.3. Electrotonic Currents

Beats with a different electrical ventricular activation order than the reference beat, e.g., a heartbeat with an incidental intraventricular block, a ventricular extrasystole, or a paced beat, necessarily cause different electrotonic currents. As already discussed in the introduction, due to electrotonic currents, action potentials of a certain myocardial region are influenced by action potentials of neighboring regions within the space constant (1–2 mm). The net result is that action potentials of a specific myocardial region will be influenced by action potentials from different neighboring regions if the trajectories of the depolarization or repolarization wavefronts change. To our knowledge, the influence of electrotonic effects in VG validation studies cannot be controlled. It is also difficult to assess. If differences are measured between the VGs computed from the predominant supraventricular QRST complex and computed from a non-premature extrasystole, it is likely to attribute this to electrotonic effects rather than to restitution effects.

#### 4.3.4. Mechanoelectrical Coupling

A final mechanism that may methodologically complicate a VG validation study is mechanoelectrical coupling. When measuring the VG in a QRST complex that differs from the predominant QRST complex because of a deviating ventricular activation pattern, the mechanical forces that develop as a function of time in the ventricles are different from the forces in the predominant QRST complex. Due to mechanoelectrical coupling (sensitivity of the electrical characteristics of the ion channels in the cellular membrane for varying mechanical stress), action potential morphology changes occur, and the VG will change. Again, this is difficult to measure in an epiphenomenon such as the ECG. Electrocardiographic imaging could possibly be helpful in assessing APD changes due to electrotonic effects or mechanoelectrical coupling.

### 4.4. Strengths and Limitations

Compared to previously published research, our study is of additional interest due to a number of methodological aspects in the study design:

- Our observations were made in humans, whereas several elementary studies on the VG were performed in dogs [1,4,30,31,32,33].
- In contrast to previous studies, which all had a limited amount of cases, we studied a larger number of subjects, allowing for statistical analysis.
- The relatively large amount of subjects allowed for the observation of a rich variation of prematurity, which permitted the study of prematurity as a possible relevant factor in VG variability.
- Our computations were the first in which the differences between ventricular gradients were expressed as VG difference vector magnitude rather than the VG magnitude difference. In our computations, any change in the VG, be it an increase, a decrease, or a rotation, contributes to the difference between two VG vectors.

However, there are some limitations:

- Because our study is an “experiment of nature”, the site of the ectopic pacemaker and its prematurity could not be controlled. This left us with an analysis of the pooled data of a patient group in which the individually different ectopic pacemaker sites and prematurities between patients have to replace experimentally controlled variations in ectopic pacemaker site and prematurity within subjects.
- Another limitation is the uncertainty in the baseline estimation of the ectopic beats. Because the VG is an ECG-amplitude integral, it is sensitive to baseline errors. Due to the prematurity of ectopic beats, it is not possible to accept the voltage immediately preceding that beat as an isoelectric reference (as we routinely do in sinus beats). Instead, we had to construct an interpolated baseline that was assessed with the aid of the isoelectric reference amplitudes associated with the sinus beats immediately preceding and following the ectopic beat.
- The VG of the predominant beat could be computed in an averaged beat, whereas the VG of the ectopic beat was necessarily computed in a single beat, which is more susceptible to noise. However, because of careful visual checking of the baseline assessment and excluding patients with a failed baseline, the remaining errors cannot explain the sometimes-large differences between the VGs of the predominant and ectopic beats within certain patients.
- Another limitation is the uncertainty regarding the classification of premature beats with strict LBBB configuration. We have classified these as beats of supraventricular origin with aberrant conduction (this was the case in 10 patients). However, there is a possibility that strict LBBB beats can also be generated by right ventricular ectopy. Although such extrasystoles often impress with an LBBB-like pattern, it is not known if these beats comply with the strict LBBB criteria. However, in our view, this uncertainty is not relevant to the conclusion of our study.
- A final limitation is that the VCGs were not directly recorded, e.g., by a Frank VCG-recording system, but instead were mathematically synthesized by the Kors transformation [37]. The impact of this methodology is difficult to assess, but because all comparisons were made within subjects and within only one ECG of these subjects, the influence on the outcome of our study is likely insignificant.

### 4.5. Clinical Implications and Conclusions

During the period in which the VG concept was formulated, it was considered important because of its presumed independence of ventricular activation order. In this way, the VG could help to diagnose ECG changes in a given subject as a change in either ventricular conduction or in the electrophysiological properties of the ventricular myocardium. Later, additional use of the VG in risk assessment was proposed (for an overview, see the publication by Waks and colleagues [43]). Both are useful clinical applications, but the premise of conduction independence of the ventricular gradient lacks thorough verification.

We compared, within patients, the ventricular gradient of their sinus beat and of a supraventricular normally conducted extrasystole, a supraventricular aberrantly conducted extrasystole, or a ventricular extrasystole. Ventricular gradient differences were expressed as ventricular gradient difference vector magnitudes. The prematurity of the extrasystoles was included in our analysis and interpretation. We sometimes observed large differences between the ventricular gradients of the sinus beats and those of the extrasystoles, especially when the extrasystoles had prematurity of <0.80. We concluded that the tenet of the conduction-independent ventricular gradient does not hold in this study group but also reasoned that a methodologically correct comparison of ventricular gradients would require equal heart rates or a spontaneous or stimulated ectopic beat that is not premature.

As a final remark, it should be stated that a possible invalidation of Wilson’s premise of a conduction-independent VG would not imply that the VG has no diagnostic potential in electrocardiography. Changes in the VG occurring in a clinical setting, in which the ventricular activation order is presumably not altered, may indicate pathology-associated action potential changes. An example is the VG change observed in pulmonary hypertension, caused by changes in the right-ventricular action potentials due to mechano-electrical feedback as a consequence of elevated right-ventricular pressure [44].

## Figures and Tables

**Table 1:** Breakdown of excluded ECGs.

Reason for ECG Exclusion	N	%
Insufficient context of the ectopic beat	223	86.8
Irregular rhythm preceding the ectopic beat	22	8.6
Baseline correction failure	5	1.9
Repeat ECG of the same patient	7	2.7
Total	257	100

Reasons for exclusion of 257 out of 375 initially selected ECGs. See the text in the ECG exclusion paragraph in the Methods section.

**Table 2:** Anthropomorphic characteristics of the study group (N = 118).

Sex (% Male)	66.1
Age (years)	64 ± 14 [24–92]
Height (cm)	175.4 ± 9.6 [148–199]
Weight (kg)	85.5 ± 17.4 [43–154]
BMI (kg/m^2^)	27.7 ± 4.8 [18.5–47.7]

Anthropomorphic characteristics of the study group. Data are displayed as mean ± SD [range]. BMI = body mass index.

**Table 3:** Cardiovascular diagnoses in the study group (N = 118).

Diagnosis		n	%
Coronary atherosclerotic disease		49	41.5
Healed myocardial infarction		28	23.7
Chronic heart failure		15	12.7
Cardiomyopathy		13	11.0
Hypertension		60	50.8
	Systemic	58	49.2
	Pulmonary	3	2.5
Hypertrophy		2	1.7
	Ventricular	1	0.8
	Atrial	1	0.8
Valvular disease		34	28.8
	Tricuspid	6	5.1
	Mitral	21	17.8
	Aortic	19	16.1
	Pulmonary	0	0.0
Congenital heart disease		6	5.1
Rhythm disorders		45	38.1
	Supraventricular	27	22.9
	Ventricular	26	22.0
Conduction disorders		14	11.9
	AV-nodal	4	3.4
	Intraventricular	11	9.3
		266	225.3

Cardiovascular diagnoses in the study group; these diagnostic statements summarize the pathological conditions that were present at the moment that the ECG included in this study was made. Percentages relate to the total number of patients in the study group (N = 118). More than one diagnosis may apply to a single patient. AV = atrioventricular.

**Table 4:** Clinical indication for recording the studied ECGs.

Indication	n	%
Periodic check/screening	71	60.2
Diagnostic workup	26	22.0
Preceding or following elective intervention	10	8.5
Acute cardiovascular incident	7	5.9
Unknown	4	3.4
	118	100.0

Clinical indication for making the studied ECGs.

**Table 5:** QRS durations of the predominant beats.

Origin and Conduction of Ectopic Beat		QRS Duration of the Predominant Beat (ms)				
	N	Mean	SD	Min	Max	*p* (vs. SN)
SN	6	90.0	18.0	74	122	
SA	20	103.8	24.3	84	166	0.21
V	92	108.8	22.4	68	184	0.047
All	118	107.0	22.7			

Breakdown of the QRS durations of the predominant beats for patients with normally conducted supraventricular ectopic beats (SN), aberrantly conducted supraventricular ectopic beats (SA), and ventricular ectopic beats (V).

**Table 6:** ECG interpretation by Glasgow Royal Infirmary software.

Interpretation	n	%
Normal	2	1.7
Borderline normal	31	26.3
Borderline abnormal	17	14.4
Abnormal	68	57.6
	118	100.0

ECG interpretation (degree of normality/abnormality) by Glasgow Royal Infirmary software.

**Table 7:** ECG interpretation by the Glasgow program.

Interpretative Descriptor	Total	
	n	%
ST abnormality	52	44.1
BBB/IV conduction disorder	35	29.7
Axis deviation	31	26.3
Old myocardial infarction	31	26.3
Hypertrophy	12	10.2
Low QRS voltage	7	5.9
Long QT	3	2.5
Pericarditis	0	0.0
	171	144.9

ECG interpretation by the Glasgow program. Categories of abnormalities relate to the predominant QRST complex. Together these interpretative statements cover all major interpretative classes of the Glasgow program; only pericarditis had a count of zero. Multiple interpretative descriptors may apply to one ECG. Percentages relate to the total number of patients (N = 118). BBB = bundle branch block, IV = intraventricular.

**Table 8:** Comparison of the predominant and ectopic ventricular gradient vector magnitudes.

Origin and Conduction of Ectopic Beat	$\left|\vec{VG_{p}}\right|\;(\mathrm{mV}\cdot \mathrm{ms})$		$\left|\vec{VG_{e}}\right|\;(\mathrm{mV}\cdot \mathrm{ms})$		*p* Values	$\left|\vec{\Delta VG_{ep}}\right|\;(\mathrm{mV}\cdot \mathrm{ms})$	
	Mean	SD	Mean	SD		Mean	SD
All (N = 118)	47.78	24.62	53.72	25.03		19.94	9.76
SN (N = 6)	57.01	27.46	59.50	32.77	0.66	17.83	10.83
SA (N = 20)	48.45	31.69	54.30	27.89	0.04	18.87	6.13
V (N = 92)	47.03	22.85	53.22	24.12	<0.001	20.31	10.37
	***p* Values**		***p* Values**			***p* Values**	
SA vs. SN	0.56		0.70			0.76	
V vs. SN	0.31		0.55			0.57	

Mean and standard deviations of the magnitudes of the ventricular gradient vectors of the predominant beat ($\left|\vec{VG_{p}}\right|$), of the ectopic beat ($\left|\vec{VG_{e}}\right|$), and of the difference vector ($\left|\vec{\Delta VG_{ep}}\right|$). Statistical comparisons were done with unpaired (columns) and paired (rows) two-sided T tests. SN = patients with a normally conducted supraventricular ectopic beat; SA = patients with an aberrantly conducted supraventricular ectopic beat; V = patients with a ventricular ectopic beat.

## Data Availability

Upon request, the authors are willing to consider sharing study data when compliant with our institutional regulations.

## Glossary

AP	action potential
AV	atrioventricular
BBB	bundle branch block
BMI	body mass index
ECG	electrocardiogram
GRI	Glasgow Royal Infirmary
ICD	implantable cardioverter/defibrillator
IV	intraventricular
LEADS	Leiden ECG Analysis and Decomposition Software
LBBB	Left bundle branch block
LUMC	Leiden University Medical left
VCG	vectorcardiogram
VG	ventricular gradient
$\vec{VG_{e}}$	ventricular gradient of the ectopic beat
$\vec{VG_{p}}$	ventricular gradient of the predominant beat
$\vec{\Delta VG_{ep}}$	difference vector between the ventricular gradients of the ectopic and the predominant beat

## References

[1] Wilson F.N., Macleod A.G., Barker P.S. (1931). The T Deflection of the electrocardiogram. Trans. Assoc. Am. Phys..

[2] Burch G.E., Abildskov J.A., Cronvich J.A. (1954). The spatial vectorcardiogram and mean spatial ventricular gradient in normal pregnant women. Circulation.

[3] Man S., Maan A.C., Schalij M.J., Swenne C.A. (2015). Vectorcardiographic diagnostic & prognostic information derived from the 12-lead electrocardiogram: Historical review and clinical perspective. J. Electrocardiol..

[4] Wilson F.N., Macleod A., Barker P.S., Johnston F.D. (1934). The determination and the significance of the areas of the ventricular deflections of the electrocardiogram. Am. Heart J..

[5] Burger H.C. (1957). A theoretical elucidation of the notion ventricular gradient. Am. Heart J..

[6] Draisma H.H., Schalij M.J., Van der Wall E.E., Swenne C.A. (2006). Elucidation of the spatial ventricular gradient and its link with dispersion of repolarization. Heart Rhythm..

[7] Axelsson K.-J., Gransberg L.N.E., Lundahl G., Vahedi F., Bergfeldt L. (2021). Adaptation of ventricular repolarization time following abrupt changes in heart rate: Comparisons and reproducibility of repeated atrial and ventricular pacing. Am. J. Physiol. Circ. Physiol..

[8] Geselowitz D.B. (1983). The ventricular gradient revisited: Relation to the area under the action potential. IEEE Trans. Biomed. Eng..

[9] Simonson E., Schmitt O.H., Dahl J., Fry D., Bakken E.E. (1954). The theoretical and experimental bases of the frontal plane ventricular gradient and its spatial counterpart. Am. Heart J..

[10] Angle W.D. (1960). The ventricular gradient vector and related vectors. Am. Heart J..

[11] Wilson F.N., Rosenbaum F.F., Johnston F.D. (1947). Interpretation of the ventricular complex of the electrocardiogram. Advances in Internal Medicine.

[12] White P.D. (1951). Heart Disease.

[13] Segers M., Boyadjian N. (1949). Étude critique du concept du gradient ventriculaire. Arch. Mal. Coeur..

[14] Berkun M.A., Kesselman R.H., Donoso E., Grishman A. (1956). The spatial ventricular gradient: Intermittent Wolff-Parkinson-White syndrome, intermittent left bundle branch block and ventricular premature contractions. Circulation.

[15] Grishman A., Borun E., Jaffe H.L. (1951). Spatial vectorcardiography: Technique for the simultaneous recording of the frontal, sagittal, and horizontal projections. I. Am. Heart J..

[16] Cosma J., Levy B., Pipberger H.V. (1966). The spatial ventricular gradient during alterations in the ventricular activation pathway. Am. Heart J..

[17] Curtis H.J. (1949). Action potential of heart muscle. Am. J. Physiol. Content.

[18] Woodbury L.A., Woodbury J.W., Hecht H.H. (1950). Membrane resting and action potentials of single cardiac muscle fibers. Circulation.

[19] Bass B. (1975). Restitution of the action potential in cat papillary muscle. Am. J. Physiol. Content.

[20] Franz M.R. (2003). The electrical restitution curve revisited: Steep or flat slope—Which is better?. J. Cardiovasc. Electrophysiol..

[21] Srinivasan N.T., Orini M., Simon R.B., Providência R., Khan F.Z., Segal O.R., Babu G.G., Bradley R., Rowland E., Ahsan S. (2016). Ventricular stimulus site influences dynamic dispersion of repolarization in the intact human heart. Am. J. Physiol. Circ. Physiol..

[22] Franz M.R., Swerdlow C.D., Liem L.B., Schaefer J. (1988). Cycle length dependence of human action potential duration in vivo. Effects of single extrastimuli, sudden sustained rate acceleration and deceleration, and different steady-state frequencies. J. Clin. Investig..

[23] Berger R.D. (2004). Electrical restitution hysteresis: Good memory or delayed response?. Circ. Res..

[24] Wu R., Patwardhan A. (2004). Restitution of action potential duration during sequential changes in diastolic intervals shows multimodal behavior. Circ. Res..

[25] Draisma H.H.M., Hooft van Huysduynen B., Swenne C.A., Maan A.C., Van der Wall E.E., Schalij M.J. (2005). Increased dispersion of ventricular repolarization during recovery from exercise. Comput. Cardiol..

[26] Litovsky S.H., Antzelevitch C. (1989). Rate dependence of action potential duration and refractoriness in canine ventricular endocardium differs from that of epicardium: Role of the transient outward current. J. Am. Coll. Cardiol..

[27] Scherptong R.W.C., Henkens I.R., Man S.C., Le Cessie S., Vliegen H.W., Draisma H.H.M., Maan A.C., Schalij M.J., Swenne C.A. (2008). Normal limits of the spatial QRS-T angle and ventricular gradient in 12-lead electrocardiograms of young adults: Dependence on sex and heart rate. J. Electrocardiol..

[28] Axelsson K.-J., Gransberg L., Lundahl G., Bergfeldt L. (2021). Adaptation of ventricular repolarization dispersion during heart rate increase in humans: A roller coaster process. J. Electrocardiol..

[29] Rudy Y. (2005). Electrotonic cell-cell interactions in cardiac tissue: Effects on action potential propagation and repolarization. Ann. N. Y. Acad. Sci..

[30] Lux R.L., Urie P.M., Burgess M.J., Abildskov J.A. (1980). Variability of the body surface distributions of QRS, ST-T and QRST deflection areas with varied activation sequence in dogs. Cardiovasc. Res..

[31] Abildskov J.A., Urie P., Lux R., Burgess M.J., Wyatt R. (1978). Body Surface distribution of QRST area. Adv. Cardiol..

[32] Abildskov J.A. (1976). Effects of activation sequence on the local recovery of ventricular excitability in the dog. Circ. Res..

[33] Abildskov J.A., Burgess M.J., Millar K., Roland W. (1970). New data and concepts concerning the ventricular gradient. Chest.

[34] Strauss D.G., Selvester R.H., Wagner G.S. (2011). Defining Left bundle branch block in the era of cardiac resynchronization therapy. Am. J. Cardiol..

[35] Macfarlane P.W., Devine B., Latif S., McLaughlin S., Shoat D.B., Watts M.P. (1990). Methodology of ECG interpretation in the Glasgow program. Methods Inf. Med..

[36] Draisma H.H.M., Swenne C.A., Van de Vooren H., Maan A., Hooft van Huysduynen B., Van der Wall E.E., Schalij M.J. (2005). LEADS: An interactive research oriented ECG/VCG analysis system. Comput. Cardiol..

[37] Kors J.A., Van Herpen G., Sittig A.C., Van Bemmel J.H. (1990). Reconstruction of the Frank vectorcardiogram from standard electrocardiographic leads: Diagnostic comparison of different methods. Eur. Heart J..

[38] Sandler I.A., Marriott H.J. (1965). The differential morphology of anomalous ventricular complexes of RBBB-type in lead V1; ventricular ectopy versus aberration. Circulation.

[39] Fellström B., Nordgren L. (1979). Initial vector rates in differentiation between supraventricular extrasystoles with aberration and ventricular extrasystoles. J. Electrocardiol..

[40] El Hajjaji I., Becker G., Kus T., Vinet A., Berkovitz A., Sturmer M. (2018). Novel criterion for the differential diagnosis of wide QRS complexes and wide complex tachycardia using the initial activation of QRS on leads V1 and V2: Differential diagnosis of wide QRS based on V1-V2. J. Electrocardiol..

[41] Kashou A.H., Evenson C.M., Noseworthy P.A., Muralidharan T.R., DeSimone C.V., Deshmukh A.J., Asirvatham S.J., May A.M. (2020). Differentiating wide complex tachycardias: A historical perspective. Indian Heart J..

[42] Jalife J., Stevenson W.G. (2021). Zipes and Jalife’s Cardiac Electrophysiology: From Cell to Bedside.

[43] Waks J.W., Tereshchenko L.G. (2016). Global electrical heterogeneity: A review of the spatial ventricular gradient. J. Electrocardiol..

[44] Kamphuis V.P., Haeck M.L.A., Wagner G.S., Maan A.C., Maynard C., Delgado V., Vliegen H.W., Swenne C.A. (2014). Electrocardiographic detection of right ventricular pressure overload in patients with suspected pulmonary hypertension. J. Electrocardiol..

